# Review of Recent Progress on Silicone Rubber Composites for Multifunctional Sensor Systems

**DOI:** 10.3390/polym16131841

**Published:** 2024-06-28

**Authors:** Vineet Kumar, Md. Najib Alam, Sang Shin Park

**Affiliations:** School of Mechanical Engineering, Yeungnam University, 280 Daehak-Ro, Gyeongsan 38541, Gyeongbuk, Republic of Korea; vineetfri@gmail.com (V.K.); mdnajib.alam3@gmail.com (M.N.A.)

**Keywords:** silicone rubber, multifunctional sensors, real-time monitoring, gauge factors, response time of sensors

## Abstract

The latest progress (the year 2021–2024) on multifunctional sensors based on silicone rubber is reported. These multifunctional sensors are useful for real-time monitoring through relative resistance, relative current change, and relative capacitance types. The present review contains a brief overview and literature survey on the sensors and their multifunctionalities. This contains an introduction to the different functionalities of these sensors. Following the introduction, the survey on the types of filler or rubber and their fabrication are briefly described. The coming section deals with the fabrication methodology of these composites where the sensors are integrated. The special focus on mechanical and electro-mechanical properties is discussed. Electro-mechanical properties with a special focus on response time, linearity, and gauge factor are reported. The next section of this review reports the filler dispersion and its role in influencing the properties and applications of these sensors. Finally, various types of sensors are briefly reported. These sensors are useful for monitoring human body motions, breathing activity, environment or breathing humidity, organic gas sensing, and, finally, smart textiles. Ultimately, the study summarizes the key takeaway from this review article. These conclusions are focused on the merits and demerits of the sensors and are followed by their future prospects.

## 1. Introduction

Multifunctional sensors represent the class of composite materials that go beyond the traditional functional properties [1]. These composite materials are equipped with sensing capabilities and are useful in various industrial applications [2]. The multifunctional sensors include a wide range of sensors, such as strain sensors, chemical sensors, and structural health monitoring sensors [3,4,5]. The integrated sensor minimizes weight and complexity while maximizing the sensing capacities. Kumar et al. [6] report that the multifunctional sensor gives real-time monitoring that can sense various parameters, such as human motion like finger bending or thumb pressing. Qureshi et al. [7] showed that these sensors can detect damage, fatigue, and deformation in real time, thereby assisting in timely repair. Some other studies show that a sensor-equipped elastomeric composite can act as a smart material and has useful applications in adaptive structures or active vibration control systems [8,9]. In some cases, like in Khoshmanesh et al. [10], the sensors are equipped with biomedical devices and are useful for implants and prosthetics for continuous health monitoring. Finally, these sensors help to detect and measure pollution, chemical gases, and humidity levels in the air [11,12]. There are various uses for these sensors, such as in the work performed by Dhall et al. [13]. These sensors offer versatile sensing capabilities without the use of complicated methods for real-time monitoring. These sensors are seamlessly integrated with the elastomeric composites with ensured compatibility and enhanced structural integration with the composite [14,15]. However, studies by Giordano et al. [16] show that there are still some challenges during the operation of these sensors. These challenges are reliability, durability, calibration, and accuracy of the final integrated sensor.

The composites used in developing these multifunctional sensors are advanced materials containing elastomers and fillers [17]. These elastomers are stretchable and electrically conductive fillers, which are added to make them useful for sensing properties, as studied by Kumar et al. [18] and Mehmood et al. [19]. The elastomers contain various classes of rubber materials like natural rubber, butadiene rubber, or the most frequently used silicone rubber [20,21,22,23]. The silicone rubber can be used with room temperature, low temperature, or high-temperature vulcanizing systems [24,25,26]. The reason for frequently using silicone rubber is because it has high resistance to aging, it is easy to process, and it is lightweight. Moreover, the electrically conductive fillers used are carbon nanotubes, graphene, or MXene [27,28,29]. When engineered for strain sensors, these composites exhibit the ability to detect and quantify external mechanical stimuli. The key features of these sensors are high sensitivity with short response time to external stimuli, tailorable properties, and their versatility, as studied by Li et al. [30] and Zhu et al. [31]. These rubber composites have wide-scale applications as sensors in wearable technology, robotics, prosthetics, and implants [32]. There are various advantages to using rubber composites for sensors. They are lightweight, flexible, stretchable, and cost-effective materials [33]. However, there are some challenges, like calibration and mechanical stability. Overall, these polymer composites have a unique combination of properties, versatility, and customization capabilities [34]. These features make them valuable for a wide range of applications that require accuracy and reliable sensing measurements, as reported by Ahmed et al. [35].

There are various reviews and research studies that involve investigating sensing configurations of the composites [36,37,38,39]. Majumder et al., [36] reviewed the aspects of soft electronics based on carbon materials. These review studies provide various applications of these materials, such as artificial skin, health monitoring, artificial intelligence, and the Internet of Things. In their study, these authors focus on electromagnetic interference (EMI) and threats from radiation to human health. Finally, the review study presents the advanced development for various forms of soft electronics. Alarifi [37] provides another review study of recent advancements in elastomers for engineering applications. They report a wide range of issues in the use of elastomers for purposes like tissue engineering, self-healing, and soft robotics. This review further reports aspects of various advancements like 3D printing, functional elastomers, and various engineering applications. In another review study, Dolui et al. [38] provide a review report on an overview of elastomeric composites for stimuli–response behavior. The main aim of their review study involves the development of mechanoadaptive elastomers for commercial use. Then, based on the structure–property relationship, the applications of these smart materials are reviewed for applications like soft robotics, actuators, and aerospace. Finally, Luo et al. [39] provide a review study on advancements in shape memory polymers with robust properties and various applications. Their review study focuses on shape memory polymers with detailed characteristics of self-sensing, self-healing, and self-learning prospects. Ultimately, the review study provides their use in various applications like robotics, smart textiles, biomedical devices, and, finally, wearable technologies.

Keeping the above prospects in mind, this review paper focuses on multifunctional sensors based on silicone rubber composites. This review article provides the latest literature survey from the year 2021 to 2024. The basic change in resistance, capacitance change, and relative current for the external stimuli were useful for functionalities in these sensors. Further, the present review study focuses on electro-mechanical properties that include response time, gauge factor, and linearity in composites-based strain sensors. Finally, the multifunctionality of these sensors for real-time monitoring is discussed. These include breathing sensors, human motion sensors, humidity sensors, organic gas sensing, and, finally, smart textile sensors. After concluding remarks, the future prospects of these sensing systems are briefly discussed.

## 2. Fabrication and Testing Methods

**Figure 1:**
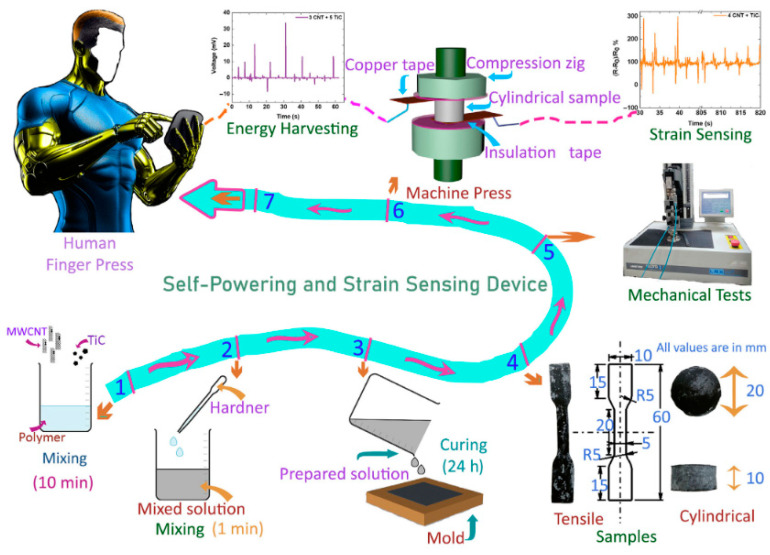
Processing steps for fabrication and testing methods. Reproduced with permission from [45].

It is well known that the fabrication method for composites is very important for obtaining target properties and required performance for applications [40]. These composite materials are made by mixing various constituents in fixed and known quantities. For example, rubber composites are fabricated by mixing rubber with fillers and vulcanizing agents as reported by Jin et al. [41]. The filler–rubber matrix mixing can be performed for a fixed time and can be dry mixing, solution mixing, or in situ polymerizations [42,43,44]. Finally, the vulcanizing agents are added, making the composite ready for the desired properties and applications. Keeping these points in mind, Manikkavel et al. [45] provide a summary of the fabrication process, including properties and final applications, in seven simple steps, presented in Figure 1. The fabrication by Manikkavel et al. [45] was performed at room temperature, and the mixing speed was maintained at 60–80 rpm. Moreover, the fabrication was performed by the solution mixing technique. They are summarized as follows:

Steps 1 and 2: Optimized mixing of a known amount of silicone rubber solution with a known amount of fillers, like titanium carbide and MWCNT, was reported. It was performed through solution mixing for 10 min. Then, the known amount of vulcanizing agent was added and mixed for 1 min before pouring them into the mold.
Steps 3 and 4: The desired shape and size of the molds were used for pouring the composite after mixing the vulcanizing agent. These molds were then pressed manually and kept at room temperature for 24 h for vulcanizing. The samples with desired dimensions were prepared and made ready for testing
Steps 5 and 6: The cylindrical and dumbbell samples were used to test mechanical and electro-mechanical properties through a universal testing machine and a multimeter. The cylindrical samples were used to study the compressive modulus. The dumbbell sample was used to study the tensile modulus, tensile strength, elongation at break, and fracture toughness.
Step 7: Finally, the real-time monitoring for strain sensors and self-powered devices was performed through finger bending, wrist bending, thumb pressing, and finger pressing.

## 3. Properties

### 3.1. Mechanical Properties

**Figure 2:**
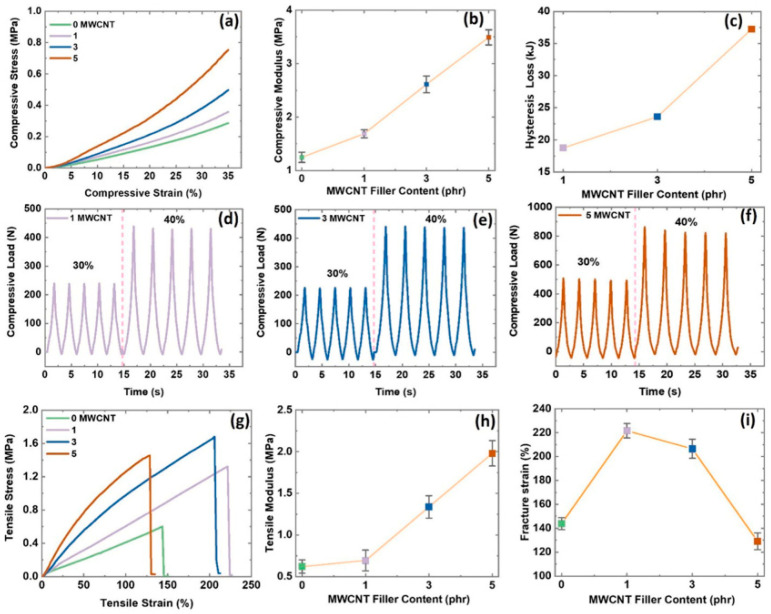
(**a**,**b**) Mechanical properties under compressive strain; (**c**–**f**) mechanical properties under compressive cyclic strain; (**g**–**i**) mechanical properties under tensile strain. Reproduced with permission from [49].

It is well known that rubber composites are versatile materials, and they have many applications. However, they are mechanically reinforced by filler particles to make them useful for applications that need high load capacity, as presented by Kumar et al. [46] and Kumar et al. [47]. Therefore, their mechanical properties are extremely important for their usefulness in multifunctional applications. These mechanical property parameters include tensile strength, modulus, elongation at break, fracture toughness, compressive strength, and, finally, abrasion resistance [48]. Manikkavel et al. [49] studied the mechanical properties of the composites and presented them in Figure 2. The results show that the mechanical properties increase with increasing filler content in the composites. These properties are compressive modulus, hysteresis losses, tensile strength, and fracture strains [50]. Figure 2a reports the stress–strain behavior of the composites under compressive strain. The results show that the compressive stress increases with increasing compressive strain and content of filler in silicon rubber-based composites [49].

The higher stress with higher strain was due to an increase in the packing fraction of the fillers used during fabrication [51]. The lower and higher packing fractions are less useful for load-bearing applications. This is due to the lower reinforcing effect of the filler at a lower packing fraction, as studied by Hu et al. [52]. However, the higher packing fraction of the fillers in the rubber matrix results from an aggregation, resulting in reduced reinforcing effects [53]. Thus, an optimum packing fraction of fillers is required to obtain the best results. This optimum packing fraction is also called the filler percolation threshold. This threshold has a higher filler content than a low packing fraction of filler but a lower filler content than a high packing fraction in rubber composite, like that by Vieira et al. [54]. Figure 2b further supports the hypothesis of filler packing fraction in rubber composites and their reinforcing effect. The results show that the modulus increases with increasing MWCNT content, reaching maximum at 5 phr loading. For example, adding the MWCNT significantly enhances the modulus, resulting in a stiff and mechanically robust composite material with good filler dispersion, as studied by Kumar et al. [55]. This good filler dispersion and efficient filler–rubber bonding are crucial for reinforcing the effect on composites. The improved interface therefore maximizes the higher load transfer and reinforcing effect by MWCNTs in the SR matrix [56]. This enhancement has multifunctional applications, offering the development of high-performance materials with high mechanical stability and durability, as studied by Zhao et al. [57].

To understand the mechanical stability under different mechanical loads, Figure 2c–f presents the behavior of compressive load under cyclic compressive strain. A higher mechanical load can be proposed due to efficient load transfer, restrained mobility, and improved interfacial adhesion [58,59]. These can be understood more briefly as (a) the higher aspect ratio of MWCNT allows them to efficiently transfer load within the composite. Under applied strain, MWCNT particles bear the stress portion resulting from the elastomer and result in enhanced stiffness, as presented by Shin et al. [60]. (b) The addition of MWCNT restricts the free mobility of polymer chains of the SR matrix. This process therefore increases the filler networking, leading to a higher modulus [61]. Moreover, the hysteresis losses were higher with increasing MWCNT content in the SR matrix. These hysteresis losses imply the dissipated energy as heat during cyclic mechanical loads. In rubber composites, this dissipation occurs due to internal friction and viscoelastic mechanisms [62,63,64]. This can be understood as the combination of elastic (recoverable) and viscous (non-recoverable) mechanisms. Under cyclic load, the energy is absorbed by the material and dissipated as heat, which leads to hysteresis losses [65]. Therefore, the researchers can gain insight into the complicated interplay among MWCNT loading and its relation to hysteresis losses. This provides a new method for the development of better composites with tailored properties, as studied by Kumar et al. [66].

Figure 2g–i provides the stress–strain, tensile strength, and fracture strain under tensile tests. The behavior of the composites shows almost the same features as under compressive tests. Here, the MWCNT plays an important role in reinforcing the silicone rubber. For example, the addition of MWCNT results in improved stiffness, strength, and electrical conductivity [67]. The main mechanism behind the improved stiffness and tensile strength is interfacial friction and filler dispersion. However, the fall in fracture strain at 5 phr is proposed due to aggregation of MWCNT and disruption of the polymer chains in silicone rubber. For example, the high content of MWCNT may result in disruption of polymer chain mobility, resulting in increased internal friction, as studied by Aguiar et al. [68] and Arrigo et al. [69], and thus a lower fracture strain at 5 phr of MWCNT. Therefore, understanding the relationship between MWCNT content and mechanical stiffness is crucial for optimizing the final characteristics of the composite. Thus, the choice of MWCNT content can be tuned to achieve the desired mechanical properties.

### 3.2. Response Time, Linearity, and Gauge Factor of Sensors Based on Composites

The response time, linearity, and gauge factors are critical for understanding how these properties measure the versatility of strain sensors [70,71,72]. Here, the response time refers to the time taken by the subjected strain sensor to respond to the external stimuli. In the composite field, it is critical for real-time monitoring and control systems. The response time has a direct correlation to the material nature, the design of the sensor, and the external environmental conditions, as reported by Pyo et al. [73]. Besides this, linearity describes the relationship between the input stimuli and the output response of the sensor. The linearity ensures an accurate and predictable sensor across the input conditions. The linear relationship is desirable, and deviations from linearity can introduce errors in sensing tests, as studied by Guo et al. [74]. Finally, the gauge factor measures the strain sensitivity of the stretchable material. It provides the change in relative resistance in response to applied strain. The higher gauge factors indicate higher sensitivity to external strain [75]. In rubber composites, the gauge factor can vary by changing dependent factors like type and content of filler, nature of elastomer, and overall stiffness of the composites.

**Figure 3:**
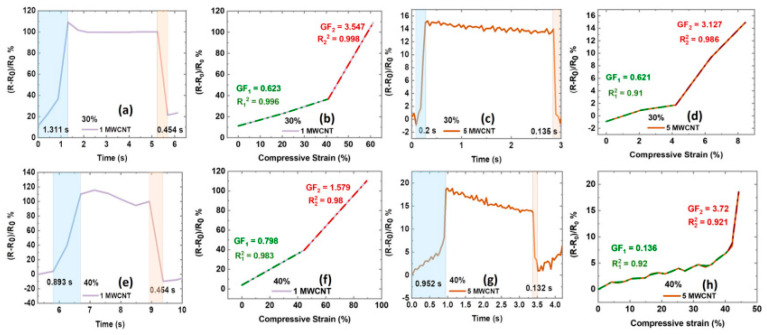
Electrical properties: (**a**,**c**,**e**,**g**) Response time under compressive strain; (**b**,**d**,**f**,**h**) Gauge factor and linearity under compressive strain. Reproduced with permission from [49].

Achieving a balance in response time, linearity, and gauge factors requires careful selection and optimization of the fabricated composite Some other factors of composites like filler dispersion, interface properties, stiffness, and structural integrity can sharply influence the overall performance of the strain sensor [76,77]. By addressing these key characteristics, the researchers can advance the versatility of composite-based sensing technologies. Keeping these points in mind, Manikkavel et al. [49] developed strain sensors based on MWCNT and silicone rubber-based composites (Figure 3). The results show that the response time was higher for the loading cycle and lower for the de-loading cycle for all composites. For example, the response time was 1.3 s during the loading cycle and 0.45 s for the unloading cycle at 1 phr of MWCNT in the composite. These features reflect the viscoelastic nature of these composites as polymer chains experience significant flow and reorganization, as reported by de Bomfim et al. [78]. Such viscoelastic nature arises from polymer chains in the rubber matrix, allowing them to flow and deform under load. The viscous behavior results in energy dissipation and a slower response time as the material accommodates the applied load [79,80].

However, such viscoelastic materials return to their original shape once the load is removed. Moreover, viscoelastic materials exhibit time-dependent responses. This feature can lead to delays in reaching equilibrium states and longer response times during loading cycles, as reported by He et al. [81]. However, upon unloading, the material undergoes a relaxation processes where stored energy is released, contributing to faster response times as the material relaxes back to its initial state. Thus, a detailed understanding of these features is essential for optimizing the performance of rubber composites for diverse applications like those studied by Huang et al. [82].

Moreover, the gauge factor increases with increasing magnitude of strain for all composites. For example, the gauge factor was 0.623 up to 40% strain and increased to 3.54 up to 60% strain. These features could be due to the reorientation of the filler particles. For example, at low strains, the filler particles may be initially oriented randomly. But, at higher strains, these particles align along the direction of strain, resulting in an enhanced piezo-resistive effect, as reported by Zhang et al. [83]. Moreover, the higher strain results in increased contact points among filler particles, resulting in an enhanced electrical conductivity and thus a higher gauge factor. Also, the higher strain can result in strain-induced crystallization that further enhances the piezo-resistive response [84]. Finally, the linearity was higher at lower MWCNT content and decreased with increasing MWCNT content. For example, it was 0.998 at 1 phr MWCNT, and it decreased to 0.921 at 5 phr MWCNT. Such features are generally complex and depend upon several factors. These factors are percolation effects, CNT dispersion, and electrical conduction mechanisms. Overall, adding MWCNT can enhance the electrical properties of the composite material. This behavior makes them promising candidates for various applications such as sensors and actuators, as reported by Alam et al. [85]. In some cases, the behavior of electrical conductivity is non-linear, especially at higher MWCNT content. This nonlinearity arises from various factors, including MWCNT agglomeration, tunneling conduction, and contact resistance between MWCNT, as reported by Kumar et al. [86]. Also, the agglomeration of MWCNTs at higher content can create localized regions of high conductivity. These features lead to non-uniform responses across the material and contribute to nonlinear behavior in composites [87].

### 3.3. Filler Dispersion and Morphology by AFM and SEM

**Figure 4:**
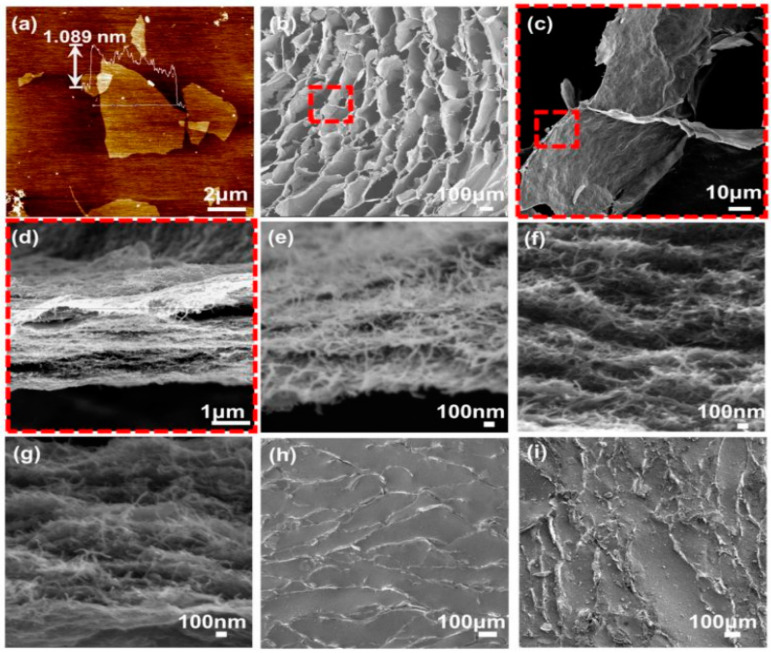
(**a**) AFM used for studying the morphology of graphene flakes; (**b**–**i**) SEM showing filler dispersion of different composites. Reproduced with permission from [92].

AFM and SEM are powerful tools that are frequently used to study the filler dispersion in composites and in a filler’s morphology [88,89]. AFM is based on the interaction forces between the AFM tip and the surface of the sample. It provides high-resolution topological information about the filler’s morphology and its dispersion state in the composite. It can reveal the distribution, aggregation, and orientation of filler particles both at the micro- and nanoscale. Moreover, AFM offers a high spatial resolution of up to atomic scale and the ability to operate in air, liquid, and vacuum, as reported by Kumar et al. [90]. On the other hand, SEM offers a focused beam of electrons to scan the surface of a composite sample. The interactions between electrons and the contact sample generate signals that create high-resolution images. Moreover, SEM is frequently used for obtaining filler dispersion, and it can visualize aggregation, particle size, and interfacial interactions in composites [91]. SEM is useful for obtaining high-resolution images of different sizes and compositions. Therefore, AFM and SEM can be useful for obtaining filler dispersion from the nanoscale to the microscale, while AFM can be useful for study at atomic scale. Therefore, Liu et al. [92] study the filler morphology using AFM and CNT dispersion using SEM microscopy, as presented in Figure 4. The reported results show that the graphene flakes used in their study have a lateral dimension of around 10 µm and a thickness of around 1 nm. These results indicate that graphene is a monolayer in nature and has a high lateral length that results in a high aspect ratio. A 1:1 ratio of graphene to CNT in Figure 4b–d shows that the fillers are well dispersed with a minimum amount of aggregates. In some cases, the CNT protrudes out from the surface of the rubber matrix, making the surface rough. Thus, proper dispersion ensures that the filler particles are uniformly distributed and leads to robust properties, as reported by Manikkavel et al. [93]. The importance of uniform filler dispersion is in enhancing load transfer uniformly and strengthening the composites. Moreover, uniform dispersion ensures the prevention of localized stress concentrations that make the composite fragile and reduce performance [94].

Figure 4e–g provides the composites with a graphene-to-CNT ratio of 1:3. As it further increases to 1:5, a sort of aggregation starts leading to lower performance of the composite. These aggregates appear due to van der Waals forces, electrostatic interactions, or surface energy effects. These filler aggregates create weak points in the composite, compromising its mechanical integrity and performance, as studied by Mora-Barrantes et al. [95]. There are various processes to improve filler dispersion, such as modification of filler or matrix surfaces and optimizing processing parameters. Therefore, well-dispersed fillers are crucial in various industries, including automotive and aerospace. Overall, composite materials with uniform filler dispersion are extensively used for lightweight, structural integrity, and functional properties [96]. Finally, Figure 4h–i shows SEM with a 1:1 ratio with in-plane and out-of-plane sections, respectively. The results show the increased surface roughness by filler addition in the composite. The rough surface is beneficial, as it promotes the interlocking of surface properties. The surface roughness results in improved surface functionality for usefulness in various engineering applications, as reported by Golhin et al. [97].

## 4. Applications

### 4.1. Real-Time Monitoring of Strain Sensor through Relative Resistance

The study on real-time monitoring sensors measures the deformation experienced by materials or given structures in real time. These types of sensors are useful for various real-time monitoring applications, as described by Rao et al. [98]. These sensors are designed to detect the magnitude of strain occurring within a material under external stimuli. These stimuli can be external mechanical deformations or changes in physical conditions like humidity, temperature, etc. These sensors are operated under various principles depending upon the application of interest [99]. These sensors can be relative resistance sensors or piezoelectric sensors. Monitoring the relative resistance change against strain magnitude is most often a strain sensor application. However, in some cases, the sensors convert mechanical strain into electrical signals resulting from piezo-electricity, as presented by Parvin et al. [100]. For example, when the sample was subjected to mechanical strain, dielectric materials like silicone rubber filled with piezoelectric materials generated an electrical charge against the applied force. These strain sensors are useful for various applications, including physical monitoring in physiology, automotive, and biomechanical contexts [101,102].

**Figure 5:**
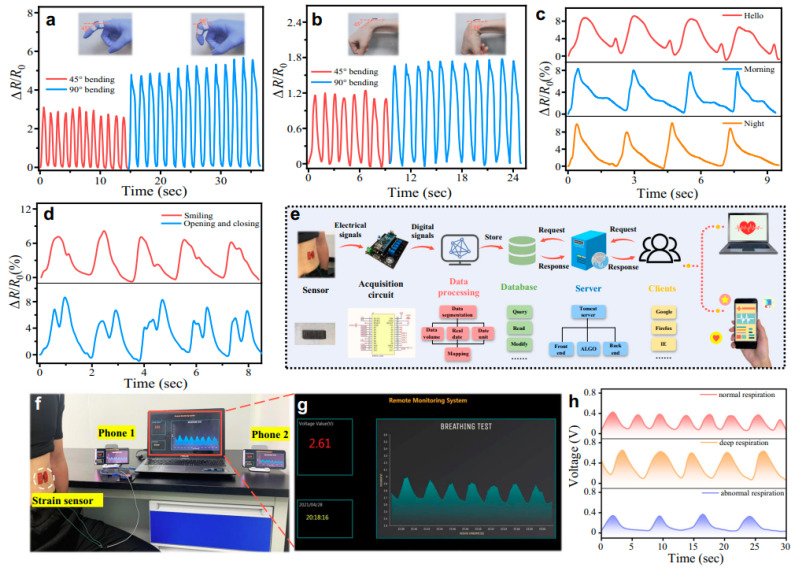
Real-time monitoring of different human motions: (**a**,**b**) Monitoring finger and wrist bending; (**c**,**d**) monitoring different word speaking; (**e**) processing the output of relative resistance change signals; (**f**–**h**) noise filtering process. Reproduced with permission from [103].

Zhou et al. [103] report the real-time monitoring of strain sensing through human motions like finger bending (Figure 5a) and wrist bending (Figure 5b). The reported results show that, in both cases, the change in relative resistance was noticed during the finger and wrist bending to 45° and 90°. The results show that the relative resistance change increases with increasing of bending angles. This can be understood between the external force exhibited by muscles when the finger and wrist bend at different angles [104]. This external force applied by the muscles results in an increase in relative resistance at a higher bending angle. As a result, this change in relative resistance of the sensor is proportional to the amount of strain experienced. The results further support that the relative resistance is stable with time, as reported by Li et al. [105]. It provides a stable real-time electrical response, better processing, and reliable output values. Therefore, the relative resistance change observed during finger and wrist bending provides valuable insights into human movements. This enables them to be used in a wide range of applications in healthcare, technology, and industries [106].

The other real-time monitoring reported by Zhou et al. involves the study of relative resistance change for speaking “Hello, morning, and night” (Figure 5c). The strain sensor developed by Zhou et al. [103] demonstrates successful monitoring of sound. The output change in relative resistance was also consistent and repeatable during the monitoring time of the sensor. Thus, the sensor enables great potential not only for speaking but also for other reliable body motions, such as finger bending or wrist bending [107]. The main mechanism involves the detection of deformations caused by sound-induced vibrations. As these sound waves interact with the sensor structure, they generate dynamic strains, and these are finally detected by the sensors as reported by Kong et al. [108]. These vocal strains result from a change in the relative resistance of the sensors. Such activities can be measured and analyzed to obtain information about the sound field.

Figure 5d presents the capture of physiological signals, like smiling, and studies the output relative to resistance concerning time. The main mechanism shows that, during a smile, the facial muscles apply external force onto the wearable sensor. This results in a change in relative resistance and transfers it into electrical signals. These signals can be processed and analyzed to differentiate between a smile and a neutral expression, as reported by Ma et al. [109]. Finally, these signals can be processed by signal processing prospects, as shown in Figure 5e–h. For example, the resistance change captured by the strain sensor is converted into an electrical response. Then, machine algorithms can be employed to interpret these electrical responses and provide reliable output data. There are various advantages to such sensors. For example, these strain sensors are a non-invasive way of monitoring various human-based activities, as presented by Wu et al. [110]. Moreover, they provide real-time monitoring, are reliable, wearable, and portable, and they can be easily integrated into clothes or skin for studying various activities in human beings. Finally, they offer a new scope for understanding and enhancing human communication and well-being [111].

### 4.2. Sensing Different Real-Time Human Monitoring through Capacitance

The study of sensing through capacitance represents a cutting-edge application for various fields that include healthcare and human–computer interactions. Simply, capacitance sensing involves measuring the relative change in capacitance that can store electrical charge [112]. These sensors have a similar working principle to piezo-resistive sensors, in that they employ a dielectric substrate separated through electrically conductive electrodes. Then, under external stimuli, the change in relative capacitance can be recorded, as studied by Liu et al. [113]. There are various real-time applications for these sensors based on capacitance. For example, monitoring real-time respiration rate, heartbeat, finger bending, or sleep monitoring. The working principle involves the study of change in relative capacitance under external stimuli [114]. By studying these variations in capacitance, the electrical signals for these physiological and human motion activities can be monitored. Overall, capacitance offers a non-invasive and versatile approach to real-time monitoring of human motions. Moreover, they promise to further enhance capabilities to improve human monitoring systems, as reported by Hong et al. [115].

**Figure 6:**
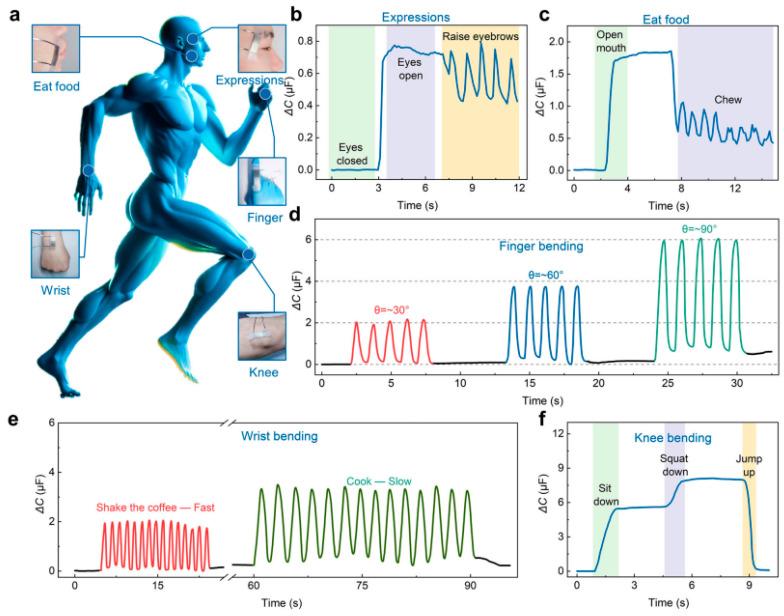
(**a**) Wearable sensors on the human body; (**b**–**f**) relative capacitance change for different human expressions and motions. Reproduced with permission from [116].

Keeping all these points in mind, Xue et al. [116] presented a high-performance sensor based on relative capacitance under external strain, as shown in Figure 6. Figure 6a shows an overview of the sensor locations on different parts of the body. The sensors show the performance from a change in expression (Figure 6b,c). This can be understood more efficiently as the change in relative capacitance under different expressions, like open–close of eyes, moving eyebrows, or eating food. For example, during eye motions or eating, the muscles exhibit mechanical force which can be measured by the attached sensor. Then, this sensor exhibits changes in relative capacitance signals in response to that force, as reported by Yuan et al. [117]. Finally, these signals can be processed and analyzed to differentiate this state from a neutral expression. Therefore, the expression strain sensing in composites enables the user to monitor structural integrity in real time, allowing for early detection of damage or deformation [118].

Similarly, the large-scale bending of human motions, such as bending the finger (Figure 6d), wrist (Figure 6e), or knees (Figure 6f), can be understood, as reported by Xue et al. [116]. The method of sensing relies on changes in capacitance to detect and quantify strains or external mechanical deformations experienced during human motion, as described by Caffrey et al. [119]. For example, the capacitance change increases with an increase in bending angle from 0° to 60° and then to 90°. This process involves gathering the magnitude and direction of the strain applied to the sensor in terms of capacitance change. Finally, the results can be processed to obtain valuable data about the motion of the wearer [120]. This strain sensing offers various merits for human motion tracking. Firstly, it can be easily integrated into lightweight wearable devices, allowing for comfortable and natural movement without any difficulty, as demonstrated by Pancham et al. [121]. Moreover, the capacitance-based sensors can be highly sensitive and responsive, with low response time and high accuracy. There are various applications for human motion strain sensing through capacitance change. These applications range from sports performance, like various human motion monitoring, to effective reality interaction and motion recognition [122]. For example, in sports, such sensors can provide a great insight into an athlete’s movements, thereby helping the trainers to optimize training programs. This will help save the athlete from injury or muscle fatigue, as studied by Tang [123]. Therefore, researchers, sports players, and health monitoring technicians can continue to innovate in this area, which will enhance human performance and patient health in hospitals.

### 4.3. Sensor for Breathing through Relative Current Change

Breathing strain sensing through current change is an innovative method to monitor the respiratory activity caused by breathing-induced strain. These sensors are based on current flow or relative current change during the inhalation or exhalation process [124]. The principle involves the relative change in current due to conductivity or resistance against external mechanical stimuli or deformations. There are various useful applications for breathing sensors, like sleep monitoring, fitness trackers, or medical diagnostics [125]. Through quantitative data, these respiratory sensors can facilitate the detection of early respiratory disorders. Thus, they can help in the prevention of respiratory diseases and improve the health and well-being of people, as reported by Li et al. [126]. There are various advantages to these respiratory sensors. For example, they can be useful, non-invasive monitors for respiratory-related aspects. They can also be useful for real-time monitoring of breathing patterns. Also, they can provide immediate feedback and insights into respiratory health. Finally, they are portable, easy to wear, and useful for clinical trials and normal health monitoring [127,128]. Therefore, breathing sensors offer versatile and convenient ways to assess breathing patterns and promote respiratory wellness.

**Figure 7:**
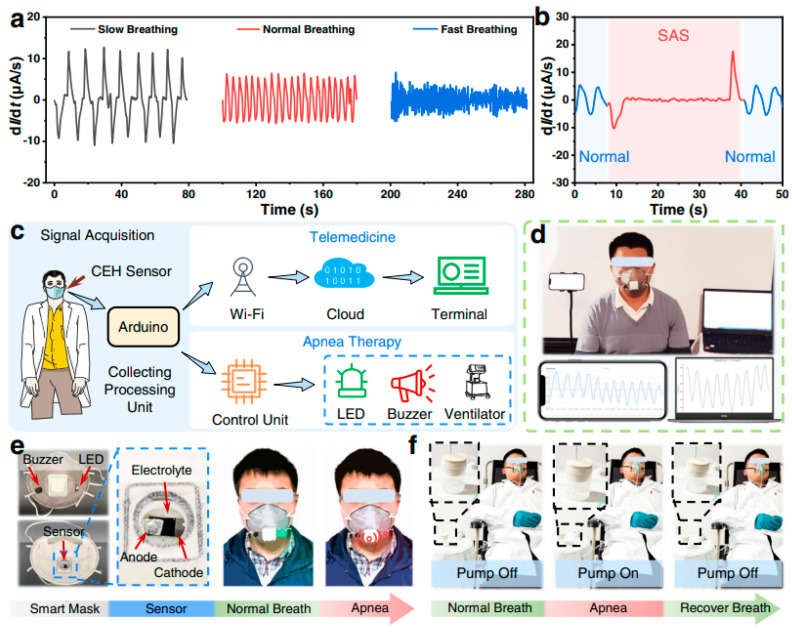
(**a**,**b**) Breathing sensor with different breathing aspects; (**c**) processing of the signal; (**d**–**f**) designed monitoring system for breathing activity. Reproduced with permission from [129].

Li et al. [129] report breath monitoring through a sensor as shown in Figure 7. Figure 7a shows the change in relative current at different respiratory frequencies. These frequencies are slow, medium, and fast, which mimics relaxed, normal, and fast breathing during exercise. These sensors rely on changes in relative current during breathing monitoring [130]. During breathing, the subtle movements of the chest or abdomen cause a change in the relative current of the sensor. These changes can be monitored by changes in relative current, which correlates with respiratory activity, as demonstrated by Sun et al. [131]. The breathing monitoring was further examined by Li et al. [129] through SAS, and the results are shown in Figure 7b. The monitored sensors recorded normal breathing as blue and the apnea process as red. The change in relative current easily shows that it is higher for normal breathing while almost zero when the testing person has apnea. Thus, the real-time monitoring shows the diagnosis of apnea.

Figure 7c,d show the designed monitoring system for breathing activity. The breathing monitoring involves the signal collecting system, a signal processing unit (Arduino), and a signal transmitting unit. Finally, an output unit (LEDs, a buzzer, and an air pump) was used as presented in Figure 7e,f. The blinking frequency of LEDs increases with increasing respiratory frequency. Thus, the system can predict the physical state of a person, such as a relaxed state or a tense state. Moreover, the real-time monitoring system clarifies if a person has apnea. For example, the LED will emit red, and the buzzer will show signals of alarms and help people take precautions to avoid dangers, as described by Vanegas et al. [132] and Nicolò et al. [133]. Finally, when the apnea was found, the air pump started working. It was followed by the patient’s active breathing, and the air pump was automatically turned off. Moreover, these sensors have various advantages, like being non-invasive, comfortable to wear, having real-time monitoring capability, and being easy to wear during their use. However, calibration, accuracy, and noise problems during monitoring are some challenges. Therefore, more novel methods are required to obtain a more efficient breathing sensor [134,135]. The diverse applications of these sensors are healthcare, sleep monitoring, and wellness. These highlight their significance in improving respiratory health and overall quality of life.

### 4.4. Sensor for Monitoring under Different Humidity Conditions

Humidity monitoring sensors are frequently used for applications that are sensitive to environmental humidity. They play a vital role in studying sectors like agriculture, industrial processes, or metrology, where humidity control is critical, as reported by Tekcin et al. [136]. For example, the industrial process that develops medicine needs precise humidity control. Thus, these sensors provide a critical role in maintaining the quality of the final product. These humidity sensors are also used in agriculture for monitoring greenhouse environments, soil moisture levels, or crop storage to prevent them from rotting due to excess humidity.

**Figure 8:**
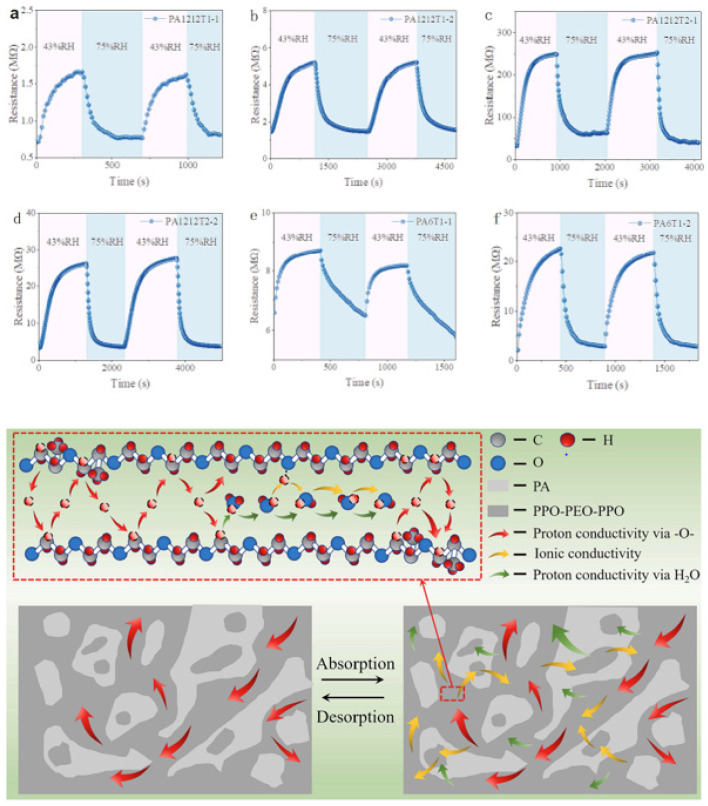
(**a**–**f**) Various behaviors of humidity sensors for different samples and mechanism of the absorption–desorption phenomena. Reproduced with permission from [141].

Finally, meteorology stations employ these humidity sensors to measure humidity levels, as studied by Thalman et al. [137]. These data are useful for weather forecasting, such as in predicting fog or evaporation processes. These sensors are based on various principles such as capacitive sensors, resistive sensors, or optical sensors. There are various advantages to these humidity sensors, such as their precision, versatility, and reliability for robust monitoring, as reported by Lee et al. [138]. Their versatility, accuracy, and reliability make them essential for achieving precise humidity control. This process helps in enhancing productivity, comfort, and safety across various industries and applications. However, calibration and air contaminants may still be challenges for such sensors [139,140]. Chen et al. [141] propose a fully functional, flexible humidity sensor for breath monitoring in Figure 8. The reported results show that the change in resistance was monitored for different humidity conditions. The graphs show the results of switching the humidity conditions from 43% to 75% relative humidity. All the samples tested for different relative humidity exert swelling/de-swelling processes during the presence and absence of water vapor. During this process, the sample swelling/de-swelling continues until an equilibrium is achieved. Moreover, the response time was different for different samples tested concerning the exposure to humidity switching [141,142]. This response time was in the range of 130 to 420 s, while the recovery time was in the range of 140 to 700 s. The absorption/desorption process and its mechanism can be understood from the below schematic in Figure 8. Based on sensors for monitoring humidity, these systems can be perfectly useful for breath monitoring. Even though the response time and recovery time are longer, they can be easily applicable for breath monitoring, as reported by Dai et al. [143]. They are also capable of monitoring normal, or fast nose breathing processes.

Monitoring breathing under different humidity conditions is very important for various medical applications. This sensing involves monitoring of medical conditions, and changes in the humidity can influence respiratory functions [144]. These functions involve respiratory rate, breath flow, and respiratory patterns. These patterns include the number of breaths per minute, the rate at which the air moves in and out of the body, etc. The breathing sensors can be of various types, like pressure sensors or flow sensors, as detailed by Amit et al. [145]. The pressure sensors involve the detection of changes in air pressure caused by breathing movements. Moreover, the flow sensors involve the testing of the rate of airflow during breathing. These processes are mainly monitored by respiratory masks. However, there are various challenges in humid environments for breath monitoring [146]. These are sensor stability, the condensation process, the compatibility of the material used during fabrication, and, finally, the comfort of wear. These breathing sensors can be useful in medical aspects, healthcare, sports and fitness, and environmental monitoring, as studied by Meena et al. [147].

### 4.5. Sensor for Different Organic Gas Sensing

Organic gas sensing refers to the detection or quantification of volatile organic gas based on polymer composite sensors. These sensors are expected to be very useful for environmental safety, healthcare, and the well-being of society, as described by Saxena et al. [148]. The key features of these chemical sensors include selective gas detection, sensitivity toward particular gases, and real-time monitoring. This real-time monitoring includes examining gas concentrations and providing timely information for safety and quality control [149]. The main mechanisms for such sensing include the gas sensing fillers and the polymer matrix. The gas-sensitive fillers are metal oxides like zinc oxide, conductive polymers like polyaniline, and carbon-based materials like CNT and graphene [150,151,152]. These fillers can change in resistance in the presence of organic gas, thereby enabling gas detection. Similarly, the polymers serve as flexible hosts for these gas-sensitive fillers. Moreover, they provide support and facilitate the gas diffusion process. These polymers are elastomers like polyurethane and other polymers like polyvinyl chloride [153,154]. The fabrication of these polymer composites includes the optimization solution mixing of fillers with polymer matrix. This fabrication process assists in achieving uniform dispersion of fillers in the polymer matrix. These polymer composites have a vast scope of applications, such as environment monitoring, industrial safety, and healthcare, as described by Zheng et al. [155] and Banga et al. [156]. These sensors have various advantages like flexibility, cost-effectiveness, and miniaturization. However, there are some challenges, like long response time, selectivity, and long-term stability.

**Figure 9:**
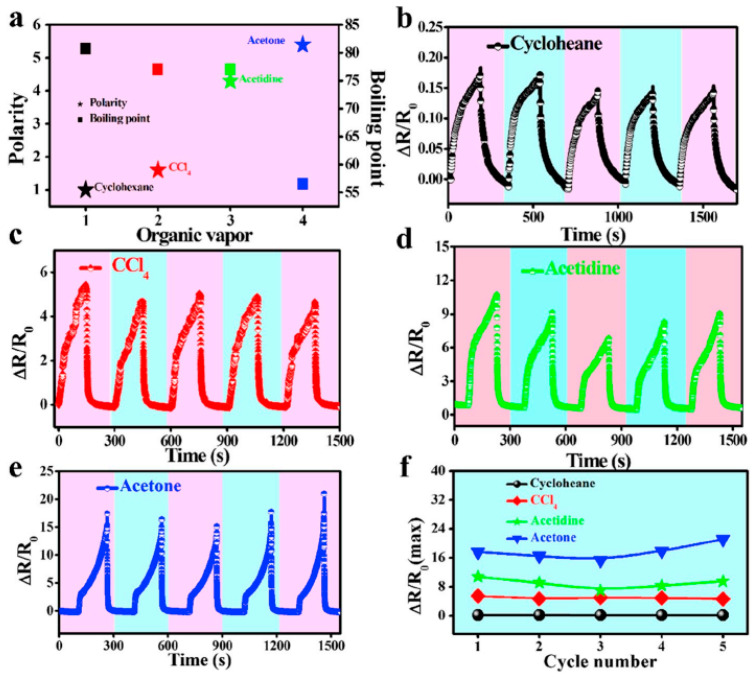
(**a**) Polarity and boiling points of different organic gas sensors; (**b**–**e**) gas sensing under different solvents; (**f**) relative resistance change for gas sensing with different solvents. Reproduced with permission from [157].

Jia et al. [157] presented the gas-sensitive properties of the composites-based sensor in the presence of organic gases. The results reported that the sensor was saturated with steam before monitoring its sensitivity with organic gas. Once the surface is saturated, the composite swells gradually and causes a change in the relative resistance of the composites-based sensor. Figure 9a shows the polarity change for exposure of different sensors. These are 0.1 for cyclohexane, 4.3 for acetidine, and 5.4 for acetone. However, the boiling point of these materials tended to be inversely proportional to their polarity change, and acetone was found to have the lowest boiling point. Similarly, Figure 9b–e reports the sensing behavior of the composites-based sensors under different organic vapors. The result clearly shows that the change in relative resistance was lowest for cyclohexane, which has low polarity and a high boiling point. For example, the relative resistance change was 0.15, 5, 8, and 16 for different solvents investigated by Jia et al. [157]. Similarly, the relative resistance change was highest for acetone, which has high polarity and a low boiling point. Therefore, it can be concluded that the resistance change is correlated with both the polarity and boiling point of the solvent’s exposure. Figure 9f shows the potential of the sensors for different solvents as a function of relative resistance and number of cycles. As shown in Figure 9b–e, the relative resistance change is dependent upon the nature of the solvent. These features are proposed due to the different polarity and boiling points of the solvents. Hence, the results provide a strong foundation for choosing the solvent with the right polarity and boiling point to obtain a better gas-sensing ability.

### 4.6. Wearable Smart Textile Sensor

The wearable sensor integrated with textiles, often termed a “smart textile”, is in focus during this decade. These smart textiles are the intersection of fashion, technology, and healthcare [158]. The incorporated sensors assist in monitoring various aspects related to human physiology. These smart textiles with integrated sensors offer comfort and flexibility, and they are thus better than traditional clothes, as presented by Hou et al. [159]. The mechanisms of smart function in textiles include electrically conductive fibers and microelectronics. For example, these sensors are integrated with the textile, and their good electrical conductivity helps in monitoring [160]. These monitoring functions include the change in resistance and the change in capacitance offered due to the stretching and bending of induced human motions. There are various applications for these smart textiles [161]. These include health monitoring, sports and fitness, and fashion and lifestyle. For example, smart textiles can monitor vital signals like heartbeat, respiration rate, body temperature, etc. These functionalities assist in providing valuable data for human beings during sports or fitness activities, as reported by Yang et al. [162]. Despite these potential functions, smart textiles with integrated sensors face challenges like durability, power management, and accuracy. Therefore, new materials and fabrication techniques are in focus to overcome these challenges [163].

**Figure 10:**
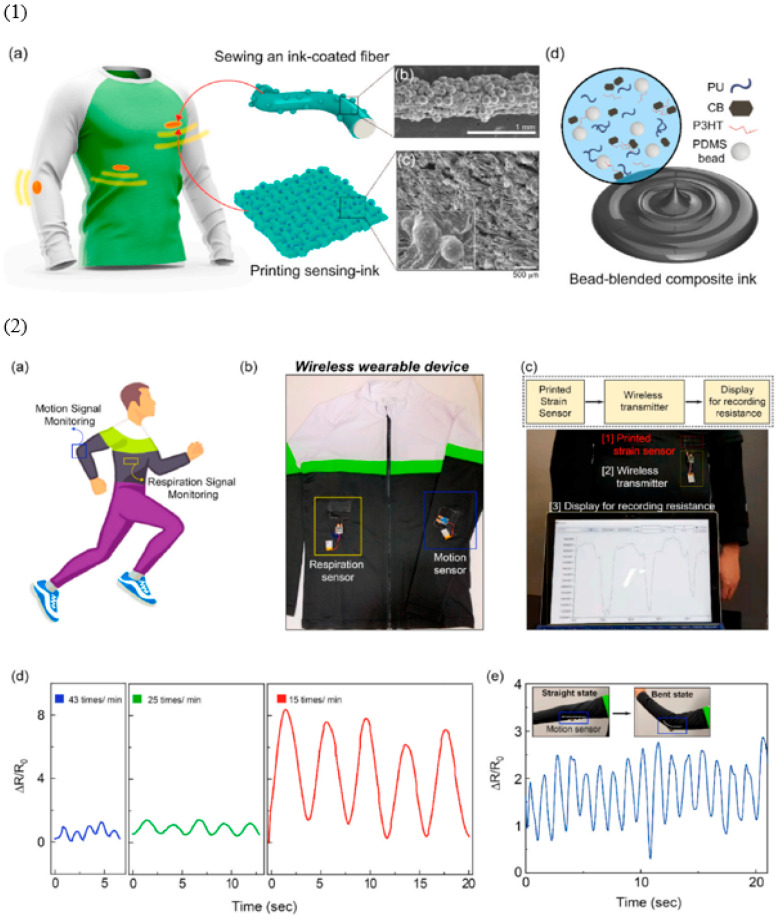
(**1a**–**1d**) Fabrication process of smart textiles; (**2a**–**2e**) real-time monitoring of human motions and respiration sensors in smart textiles. Reproduced with permission from [164].

Keeping the above points in mind, Jang et al. [164] studied smart textiles, as shown in Figure 10. Figure 10(1a–1d) shows the fabrication of smart textiles and their microstructures using SEM microscopy. The electrically conductive ink, based on carbon black, was blended with the textile fiber to make the fiber electrically conductive. When this conductive ink is blended with textile fibers, it will form electrically conductive pathways, thereby allowing the electric signals to transmit throughout the integrated sensor [165,166]. This carbon black was mixed with binder materials like elastomers to form printable or coating-ready formulations. For example, the blended composite ink by Pang et al. [164] contains polyurethane, PDMS as elastomers, and other necessary additives. Then, the smart functionalities of these resulting smart fabrics are reported. Similarly, Figure 10(2a–2e) by Jang et al. [164] shows the integrated smart sensor on textiles for real-time monitoring experiments. The results show the relative resistance change for different human motions using motion sensors or respiratory sensors. The output signals in relative resistance change printed on the textile are obtained through wireless connections. These signals are sent to a wireless transmitter and display the recorded change in relative resistance against strain through external stimuli [167,168]. Finally, real-time monitoring through motion sensors and respiration sensors can be achieved. The results show that the relative resistance was sensitive to the frequency of respiration and the magnitude of human motion, as reported by Bidsorkhi et al. [169]. Overall, some smart functionalities of these smart textiles include electrically conductive pathways, flexibility and stretchability through elastomers, washing ability, and durability. However, some challenges need to be overcome to fully explore the potential of these smart textiles [170,171].

Table 1 summarize the multifunctionality of the composites and is discussed below.

## 5. Conclusions

The present review provides a detailed overview of the latest studies on multifunctional sensors. The elastomeric matrix, like silicone rubber, was found to be a promising rubber matrix due to its dielectric and easy processing features. The use of silicone rubber is not only useful to obtain robust composites but also to make them useful for various sensing applications. These applications are sensing breathing activity, environment or breathing humidity sensors, organic gas sensing, and smart textile sensors. The versatility of these sensors is due to the excellent properties exhibited by the composites based on silicone rubber. Moreover, electrically conductive fillers like carbon black, graphene, or carbon nanotubes make these composites potentially useful for wearable electronics applications. These fillers improve the response time and assist in achieving a high gauge factor and great linearity. Such high performance after adding these fillers in silicone rubber is due to the high electrical conductivity, high modulus, and high surface area of these nanofillers.

### Current Challenges and Future Prospects

The mechanical properties of sensors play a crucial role in their overall performance, affecting their sensitivity, accuracy, reliability, and lifespan. There are various benefits, such as enhanced sensitivity, improved accuracy, durability, lightweight, and wide processing range due to the good mechanical stretchability of rubber composites. These can be detailed as (a) the enhanced sensitivity involves the right selection of materials during fabrication with good piezoelectric or piezoresistive coefficients. Moreover, achieving good stiffness helps in achieving a more pronounced sensor response to small mechanical deformation or external stimuli. (b) High dimensional stability and high precision manufacturing performance assist in producing sensors with consistent performance and reliable results. (c) High fatigue resistance and good wear resistance can assist the sensor to withstand extreme environments, making them suitable for high-durability products. However, there are some challenges as well. For example, complexity, high cost, environmental sensitivity, low power density, and miniaturization limits. These can be detailed as (a) the high-performance materials with excellent sensitivity can be expensive, thereby increasing the overall cost. Moreover, materials that offer high sensitivity and high stiffness can be brittle and prone to cracking under continuous cyclic mechanical deformation. (b) Extreme environments, like high temperature, humidity, or corrosion, can alter the mechanical properties and reduce the sensor performance. (c) Miniaturization limits and maintaining mechanical structural integrity are difficult for long-term operations and thus affect the overall performance of the sensor. Therefore, balancing the mechanical properties to the optimum level is desired to obtain a robust sensor.

The wide use of silicone rubber for real-time sensor applications is well known. The use of silicone rubber in such applications is mostly because of its ease of processing, simple vulcanization steps, low hardness, biocompatibility, and wide operational temperatures. However, various challenges can affect the sensor performance when using silicone rubber. These challenges are low mechanical stability and poor durability, sensitivity to the environment, signal interference and noise, electrical performance challenges, and difficult calibration. These challenges can be understood more briefly as (a) they are prone to degradation under fatigue tests, especially for applications involving repeated flexing or stretching. (b) Although silicone rubber has a wide operational temperature range, prolonged exposure to extreme temperatures can still affect its mechanical properties, potentially influencing the sensor’s performance. (c) The mechanical hysteresis loss in silicone rubber during fatigue tests can lead to reduced precision and reliability of measurements. (d) Achieving high electrical conductivity can be challenging for silicone rubber-based sensors. The conductive pathways in silicone rubber can degrade, especially under mechanical stress, leading to reduced sensor performance.

Medical-grade silicone rubber is widely accepted for medical applications in sensor fabrication and medical implants. However, several factors are accounted for while evaluating the toxicity of the silicone rubber composites used in fabricating sensors. These are: (a) the non-toxic nature of silicone rubber is compromised by adding fillers and other toxic ingredients to make it stiff and may add toxicity. (b) The silicone rubber can degrade in extreme environments. Extreme weather makes the silicone rubber release toxic volatile compounds, thereby making them toxic. (c) Various catalysts are used during the fabrication of silicone rubber products. The residues from these catalysts might be toxic if they are not fully reacted or adequately removed during processing. Finally, (d) proper disposal and recycling practices should be followed to prevent environmental contamination by toxic substances that may be present in silicone rubber composites. Therefore, the toxicity of these silicone rubber-based composites can be influenced by the presence of additives, fillers, degradation products, and manufacturing residues and must be considered before using them for a particular application. In addition to the toxicity, the antibacterial prospects are very important for silicone rubber-based composites. Notable researchers such as S. Sarraj et al. [172,173], K. Mazur et al. [174,175], B. Fellice et al. [176,177], and R. Yamashita et al. [178] provide significant contributions in this regard.

Graphene and carbon nanotubes are in great demand for obtaining multifunctional sensors because of their outstanding properties. Thus, the future prospects of silicone rubber and carbon nanofillers-based composites have bright prospects for multifunctional sensors. This review study found that electrically conductive silicone rubber-based composites can be tailored to have various properties. These properties are flexibility, durability, resistance, and extreme temperature stability. The nature of these composites makes them potentially viable for a wide range of sensing applications. The multifunctionality of these composites can be engineered to sense pressure, strain, humidity, breathing, or temperature. These sensors can be integrated into various devices and systems, including wearable technology, Internet of Things devices, and medical implants. The other benefits of these composites, like being lightweight, sustainable, environmentally friendly, and easy to fabricate, help in achieving large-scale production for industrial use. Moreover, these composites are mostly biocompatible due to low toxicity and thus useful for medical implants and other health monitoring applications like breathing sensors. The use of silicone rubber and carbon-based nanofillers is also sustainable, as they are resistant to moisture, chemicals, and UV radiation. Therefore, these composites have a bright future for multifunctional sensors.

## Figures and Tables

**Table 1:** Summary table for the type of fillers and rubbers, fabrication technique, properties, and their respective applications.

S. No.	Type of Filler and Rubber	Fabrication Technique	Properties	Applications	Reference
1.	Multi-wall carbon nanotube, titanium carbide, and silicone rubber	Solution mixing	For the best sample, the tensile strength was 1.34 MPa, while the compressive modulus stands at 3.87 MPa	Thumb pressing exhibits a voltage of ~100 mV, while the gauge factor was 23.9.	[45]
2.	Multi-wall carbon nanotubes, and silicone rubber	Solution mixing	Hysteresis loss was 37.24 kJ, while the compressive modulus was ~180% higher than the unfilled sample	The thumb press results in a relative resistance change of 350. The gauge factor was 14.4	[49]
3.	Multiwalled carbon nanotubes, graphene, and silicone rubber	Solution mixing	Thermal conductivity was 1.3 W/m/K, which was 465% higher than the unfilled sample	Electromagnetic interference shielding effectiveness was 42 dB in K-band	[92]
4.	Graphene and silicone rubber	Solution mixing	Mechanical stretchability was 650%	The gauge factor was 1078.1, and the response time was ~140 ms	[103]
5.	Polyvinyl chloride, polydimethylsiloxane rubber, and silicone rubber	Solution mixing	The relative capacitance was up to 0.75 µF for different human motions	The gauge factor was 9.1 × 10^6^, the linearity was 0.9997, and the response time was 17 ms	[116]
6.	Graphene oxide, silk fibroin, and lithium bromide	Solution mixing	The change in current was ~10 µA/s for slow breathing and ~3.5 µA/s for fast breathing	Sensitivity of 0.09 µA/s/1%, response time of 1.05 s, and recovery time of 0.08 s	[129]
7.	Thermoplasticpolyamide elastomer,	Heat-melt method	The resistance was in the MΩ range and decreased with increasing relative humidity for all composite samples	Sensor linearity was 0.99, and response time was 4 s	[141]
8.	Polydopamine, thermoplastic polyurethane mats, and reduced graphene oxide	Solution mixing	Tensile strength of ~7 MPa, elongation at break of ~600%	The gauge factor was 185, and the response time of 100 ms	[157]
9.	Silicone rubber, polyvinyl alcohol	Solution mixing	Tensile strength of ~9 MPa, and elongation at break of ~500%	Gauge factor was 57, wide sensing range was ~130%, excellent repeatability of >10,000, and waterproof with a contact angle of ~112°.	[164]

## Data Availability

Not Available.
